# Academy of Stomatology

**Published:** 1903-07

**Authors:** 

**Affiliations:** Academy of Stomatology


### Reports of Society Meetings.

### ACADEMY OF STOMATOLOGY.

A REGULAR meeting of the Academy of Stomatology was held at its rooms, 1731 Chestnut Street, Philadelphia, on the evening of Tuesday, January 27, 1903, the President, Dr. R. Hamill D. Swing, in the chair.

A paper entitled "Porcelain Inlays and Restorations" was read by Dr. W. T. Reeves, of Chicago, Ill.

(For Dr. Reeves's paper, see page 489.)

### DISCUSSION.

*Dr. Joseph Head.*—The innumerable good points of Dr. Reeves's paper speak for themselves, and as I am well known to be in hearty sympathy with him on the subject of porcelain inlays, I shall not therefore take up valuable time in discussing the points upon which we are agreed, but rather the points wherein we differ. When he criticises the Western dentist who claims that he is able to prepare a cavity and insert a porcelain inlay inside of an hour as doing crude and inartistic work, I can only say that I have frequently prepared two cavities and put in two porcelain inlays inside of one hour. By so confessing this, I no doubt lay myself open to the same charge of doing inartistic work, but as much of this work has lasted for five or more years and in many instances is not apparent at a distance of ten or fifteen inches from the patient's mouth, I am compelled to feel that for all practical purposes it fulfils its end. When Dr. Reeves says that it takes him two or three times the period of an hour to make a filling, I am sure that he has over-estimated the difficulty of the work. Some fillings of exceptional difficulty would demand so much time, but the ordinary approximal or labial inlay can be readily finished inside of the period of an hour. He has confessed this in a later portion of his paper, where he says that he can make an inlay much more expeditiously than the average operator could put in an ordinary gold filling, pin-head fillings excepted; therefore, I should like to ask Dr. Reeves which of these statements he wishes us to understand. Does he mean that he can make inlays more rapidly than the ordinary gold filling can be inserted, or does he mean that it really takes him two or three times the space of an hour to make the ordinary inlay?

Concerning his enumeration of conditions where gold could not be used and where porcelain could, I am constrained to say that while I agree with him in many instances, in others I disagree. He says that he can do permanent work with porcelain where it is physically impossible to prepare a cavity even for a cement filling, which, of course, is a slip of his pen; for, as porcelain has to be put in with cement and certainly requires a much more careful preparation of the cavity than a cement filling would ordinarily, it is difficult to see how the extra work required for preparing the cavity for porcelain can be done, where the same work would not be possible in the preparation of a cavity for cement.

Concerning his remarks upon compatibility, in which he claims that porcelain lasts through some intrinsic quality of compatibility with tooth-structure, and states that it is much more advantageous than the compatibility of gold, I should like him to make clear to us why it is that gold inlays properly cemented into place will last quite as well as porcelain inlays. Does he not mean that the cement properly protected is the great tooth-saver? That the cement that will wash out of an unprotected cement filling will when protected either by gold or porcelain make an edge that is practically immune to decay?

Concerning his method of getting the color, I have only words of praise, although I hardly feel that he has mastered the shadow problem thoroughly as yet, and I should be very glad to see work that would not allow variation in shadow according to the direction in which the light was thrown upon it. His plan of having two separate colors in the same inlay, one to represent the enamel and the other to represent the dentine in inlays of considerable size, is a most excellent plan, and I have frequently taken advantage of some such procedure by making my labial cavity fillings the color of the enamel only, and relying upon the yellow cement underneath to give the color of the dentine which shines through the surface of the tooth at the cervical margin. However, as Dr. Reeves well must know, porcelain inlay work contains so much of the artistic problem that specific methods of obtaining the color can no more be laid down than an artist could lay down specific rules for obtaining the proper color in the portrait he is painting. But each method is of value, and the remarks of Dr. Reeves on this subject should be received with the thanks of the profession, and I for one am heartily obliged to him for throwing light on this most difficult question.

Concerning his remarks on the matching of discoloration in the tooth, I am not in a position to speak, for I bleach the enamel before I put in the inlay, and, therefore, have practically no stains to match.

I have not had perfect success in using the Brewster bodies, but since Dr. Reeves uses them with such good effect, I, of course, am to blame. In my hands the Whiteley bodies and the porcelain of the S. S. White Company have given the best results.

The plan that Dr. Reeves uses, of burnishing his matrix three separate times before it is finished, would in my hands not give such perfect edges as a single burnish. The platinum so treated by me would be apt to act like an overswaged plate, the edges rising up from the centre. When I burnish the ordinary matrix into place, the metal should never budge from the cavity walls until it has been spun into place from the margins to the bottom of the cavity; however, this is a mere matter of detail. Some gentlemen can work better by taking an impression of the cavity and swaging the matrix with the die and counter-die. Others have the double burnish, the second one being given after a portion of the porcelain has been baked in the matrix. Dr. Reeves uses three separate burnishings, which necessitates three separate annealings of the platinum.

Before closing, I must say that Dr. Reeves's interpretation of the physical law of adhesion does not seem to me so conclusive as to make me give up undercutting the inlay and undercutting slightly the cavity walls. The adhesion of the cement is a great source of strength in supporting the porcelain as well as the tooth-structure, but slight undercuts can do no harm and may do a great deal of good.

However, his good results speak for the value of his method, and therefore, I can only congratulate him upon his procedure, and I can also congratulate the profession, for in the multiplicity of good methods lies the hope that the perfect method will in time be given to us.

I also cannot see how the porcelain is a saver of the pulp, since the porcelain is protected from the tooth-structure by the cement.

*Dr. E. T. Darby.*—Through the courtesy of Dr. Reeves I had an opportunity of reading his paper before he came on from Chicago, and I was intensely interested in it, as I have been to-night in hearing it read.

Dr. Reeves's work speaks for itself. He is undoubtedly one of the very best porcelain inlay workers in our country. I have never seen more beautiful work than that exhibited by him last summer at Niagara Falls. I have been somewhat familiar with porcelain work for a number of years, and with the work of Jenkins in Dresden. I saw him do first the porcelain work with the low-fusing body, and was much pleased with his results. I have seen a good deal of his work that has come to this country in the mouths of my own patients, and while I have been pleased with the results he produces, I cannot say that the work he did is any finer than that I have seen done in this country by Dr. Reeves and Dr. Head.

The question of high- and low-fusing bodies is a very important one. The objection on the part of the advocates of the high-fusing body is that low-fusing body changes its shape and color. My experience and observation have taught me that the low-fusing bodies give a much more beautiful surface and that the inlays are smoother. That low-fusing bodies do sometimes change I admit. I have seen instances in which the color has changed and the surface lost its beautiful polish.

As for the technique of the two methods, that used with the high-fusing bodies is preferable as a time-saver. If gold be used as in the low-fusing, it is necessary to invest the matrix before baking the inlay. Dr. Jenkins claims that the matrix retains its shape better by using No. 30 gold and investing the matrix than could be secured by one one-thousandth platinum and not investing.

I have of late rather switched off from the low-fusing bodies, and have been using the Whiteley body with satisfactory results. I do not, however, think the inlays are as beautiful when first made as are those made with the Jenkins body.

One point to which possibly exception should be taken has already been referred to by Dr. Head, that is, that the inlay is held in better position by not making undercuts than by making them. Another point is that the cement does have strength and holds the materials together.

Dr. Reeves says that he does not extend his cavity margins, and that these surfaces are immune to decay because filled with porcelain. With this I cannot agree. I think that if the cavity is not properly cleansed decay will recur around the porcelain filling. I do not believe the cavity is immune because filled with porcelain. I should extend my margins well and should not object to labial extension if porcelain be used, because it is not unsightly. I would prefer to extend my margins to avoid recurrence of decay rather than to do as Dr. Reeves advocates.

*Dr. V. T. Smith.*—My experience with porcelain inlays has not extended over a great number of years, but the work has been of great interest to me. I think Dr. Reeves has presented the subject in the most scientific and thorough way possible with the present facilities. I consider myself very fortunate to be able to witness his operations. There are, however, a few exceptions which I would take. It would be better if inlay workers knew exactly when to carry their margins beyond all points of contact. The cervical borders are sometimes apt to be overlapped by the less careful operators.

Many of the profession labor under the impression that the Jenkins system is the same as that which came out originally. The fusing-point has been raised and the material is better. One particular advantage is the close adaptation obtainable with the gold matrix. If there are any folds or hard places the gold will burnish down very much better and give closer margins than the platinum. I have, however, been using to a large extent the high-fusing bodies and think that they have a great many advantages. I do not mean to speak altogether in favor of the low-fusing bodies, but to let the members know that there has been a difference.

I was very glad to hear the matter of retention brought up. My experience has been that there should be as much retention in the cavity walls as possible to go without the making of undercuts. I believe that Dr. Reeves's plan of using hydrofluoric acid is an excellent one. I think the acid is more useful than the grooves to hold the inlay in place because you have adhesion over the entire body of the inlay; and in the other method it is present only in the grooves. Since I have been using the hydrofluoric acid method I know of no inlay which has come out of its place, except in several cases in which I am positive the occlusion was allowed to interfere. I would like Dr. Reeves to explain his method of using the acid, as a matter of interest to the members.

*Dr. Hornberger,* New York.—I have thoroughly enjoyed both the paper of Dr. Reeves and the demonstration given by him this afternoon. I belong to the other faction: I am a Jenkins man. I cannot say that I have tabooed all the other methods, but I have tried them all, and have come back to the Jenkins method. I use platinum in the majority of cases because it is a time-saver. I can get better color and more natural-looking inlays by using the Jenkins body than with the others; that is to say, with a great saving of time. Dr. Reeves said that one reason inlays have to be ground is because the low-fusing body balls up. That is the case if you over-fuse the body, but if the body is guarded you can get it out before it does so. If you wait until it balls you burn out some of the color. I am not in favor of any low-fusing body except the Jenkins. The latest Jenkins body is much superior to the original. I have taken a bit of the Jenkins body and stood on it with my entire weight, and it has not broken; and it has the advantage of being capable of as high a polish on its edges as on the surface. I have had a porous inlay only in case of over-fusing or when too much fluid has been mixed with the body. Then it is bound to settle down. If you will pack the body down tightly you can get it as hard as any you can use.

I personally use undercuts in my cavities, and also use grooves in the inlay. When in doubt I use in addition hydrofluoric acid. There are some cavities involving the incisal edge which I believe must have some retention in the way of pins. I do not think large fillings involving the incisal edge will remain in for any length of time without them. I have adopted the following method (illustrating on board): With a small bur I drill a pit in the cervical wall of the cavity parallel with the pulp, and then make another at a point near the cutting edge and parallel with it. Platinum wire No. 24 is given a right-angle bend and fitted to the two holes in such a manner as to leave a platinum loop slightly above the pulpal wall of the cavity. I then put cement into the pits and force this wire into it and bring the cement up under the loop so as not to have any undercuts. After that is finished I make the matrix and bake the inlay. I then use the carborundum stone to undercut the groove corresponding to the loop. I then remove the cement from beneath the loop and cement the inlay to place. The cement crystallizes around the loop and retains itself in the cavity of the porcelain inlay and cannot be pulled out.

*Dr. A. N. Gaylord.*—I feel that in inlay work there is a place for both methods. I commenced to work with the high-fusing body, and have done work with it which I considered satisfactory. I have also done work with the low-fusing which I think is satisfactory. There are places where I would prefer one and consider the other inferior, and *vice versa.* I do feel that closer adaptation is possible with the gold matrix than is possible with the platinum matrix. I have tested the gauges of No. 30 gold and $^1/_{1000}$ platinum, and find the former to register $^6/_{10000}$ of an inch on the micrometer, while the so-called $^1/_{1000}$ registered $^{16}/_{10000}$, a difference between their gauges of $^1/_{1000}$ of an inch. This may explain the better adaptation obtainable with the gold matrix. I think I can do better work, get closer adaptation, better color, and generally better results with the low-fusing body. It takes more time, but this should not be a matter for consideration. It is the result and not the time saved we should consider.

*Dr. Hornberger.*—I would like to say a word in regard to the strength of the Jenkins body. I had occasion about a year ago to make a pivot tooth for a man lifting heavy weights. At the same time I removed a defective bridge. Whenever he lifts these weights he grits his teeth together. The pivot lasted for four months under this excessive strain, and I replaced the bridge and made another pivot tooth, which has lasted for eight months and does not at present show the least trace of any crack. This shows that there are other things strong besides the high-fusing body.

*Dr. W. H. Trueman.*—I am not an enthusiastic porcelain worker. Porcelain inlays, I think, have been before the profession not fifteen years, but at least four times fifteen years. We can find record of them year by year from that time to the present. When Mr. Murphy began his work in Philadelphia in 1835 he had to fire up a large porcelain furnace, and it was only when the little convenient furnaces came into use that the work was done without great trouble. I admire very much the essayist's enthusiasm. I have always been rather conservative, and in many cases, when I have been about ready to take up a method, the profession has discarded it. I cannot understand why porcelain should be so much more compatible with tooth-structure than some other things. I cannot see why the microbes that form around gold fillings are not found around porcelain fillings. I can appreciate the benefit of glazed surfaces, but I am not so certain that such surfaces are so thoroughly compact as we sometimes think. In one instance in a porcelain tooth worn in the mouth for a long time the entire surface was found to contain a bacterial growth which extended some distance from the surface. I do not think that the porcelain inlays are any less porous; but, at the same time, I am not an expert, and recognized from what Dr. Reeves has said that he is.

I would like to know why an artificial tooth should chip and an inlay not. I have put a few in position, but have been careful that no pressure of mastication should be put upon them. I have had the same experience with crowns. If this always occurred in my own work, I would think it my own fault, but I find it in the work of others.

Now, it is all right to try these things, but I think it is just as well to go a little slow until we find out what can be done with them. Porcelain work has its place, and I think it is an excellent thing in those cases in which it is indicated. We have to recognize our own limitations.

Points have been mentioned that are new to me, but they are plausible, and I am strongly impressed that if those who, like myself, are not experts would take hold of them they would be found helpful.

I am glad to have heard the paper, and thank the doctor for having come here to read it. I know I shall carry home much that will add to my knowledge whether I make use of it or not.

*Dr. Reeves.*—I believe Dr. Trueman's results come entirely from the fact that the porcelain was ground and the glazing removed so that the porcelain absorbed the materials which were burnt out by the heat. He speaks of a crown breaking, and asks why a thin film can stand as much strain as a great bulk of porcelain. I care not how thick the inlay is, when it is cemented and the cement crystallized the film of inlay has the full strength of the tooth; whereas, a number of times the thickness in the form of a crown may not have the same strength. The inlay will stand an unlimited amount of stress.

One gentleman referred to a case in which the tooth had to be ground. In many instances on the cutting edges of the anterior teeth the inlays must be ground to provide for the wear of the natural teeth.

I think it is the exceptionally favorable case that can be completed in the time that Dr. Head states. I leave the rubber dam on for thirty minutes after the inlay is set. The cavity I spoke of was a large one involving an incisal edge.

You cannot put in a cement filling that is subject to stress in any tooth unless you provide something to hold that filling. The cutting of these undercuts to hold the cement filling is the last straw. The preparation of a cavity for inlays in these cases is the simplest possible.

Regarding the compatibility of porcelain inlays, the point was advanced that gold inlays preserve the teeth as well. I think there is a misconception of the class of cavities meant. The cavities referred to that establish the condition of compatibility of material are those so close to an exposure of the pulp that the pulp would be absolutely sure to die under any cement filling. It will be more likely to do so under gold inlays than under an all-cement filling. The compatibility comes in the condition which the porcelain brings to us. The inlay should be carried to the full depth of the cavity. The porcelain comes nearest to restoring the tooth to its normal condition.

With regard to grooving, I would say that in 1896 I used the last pin in any cutting edge or any form of cavity as a means of retention. Seven years ought to be a good length of time in which to prove the value of close adaptation. I know it is a hard thing for any dentist to get away from the idea that he must have some means of retention, but I believe that close adaptation is the secret of inlays staying in cavities.

Dr. Head spoke of the porcelain as a preserver of the pulp. I could cite numerous instances in which I had complete exposure of pulps, many of them made by accident, and in which the pulp was in a healthy condition. After putting in inlays there has been no further trouble. About nine months ago a young girl was brought to me who had broken one of her central incisors, leaving an exposure of the pulp. I washed the cavity out with carbolic acid and put a little temporary filling upon the tooth. The tooth was sensitive until the next appointment. I made an inlay, and, knowing where the pulp was exposed, I avoided burnishing over that spot. When the inlay was made and set she experienced no pain from the pressure, but a little from the heat of crystallization. In fifteen minutes after it was set she was entirely free from all pain, and has never had the slightest discomfort from the tooth since.

If I understand the argument regarding the thickness of the matrix metal, it is on the line that the metal occupies the same amount of space which is afterwards filled up with cement. In a cavity with walls at right angles to the pulpal wall the thickness of the material would come off of both sides, but when the cavity has walls at obtuse angles to the pulpal wall the thickness of the matrix makes no difference. The inlay sinks into the cavity and fits at the margins.

*Dr. Gaylord.*—I grant that that may be so in the majority of cases, but there are cavities in which you have three surfaces to deal with, and where the sides do play a part.

*Dr. Reeves.*—Well, I have made them of all kinds, and have yet to find places where the matrix makes any difference at all so far as taking up space is concerned. I think it is close adaptation that will give strength of retention.

*Dr. Hornberger.*—May I say, quoting from your own paper, that a film of the porcelain, no matter how thick or how thin, provided you have crystallized cement behind, becomes strong. Consequently, if, as in the case I referred to, you have an inlay with a cavity in the centre and have crystallized cement in that central cavity, it must be strong.

*Dr. Reeves.*—But you have not got cement all around your pin where it enters the tooth; you have porcelain around your tooth.

*Dr. Hornberger.*—I undercut the central cavity so that the inside is larger than the outside. Consequently the cement must go in and the pin is cemented into the tooth.

*Dr. Reeves.*—I did not understand your method; I supposed you had a baked-in pin. There will be one objection to that method. In cavities approaching close to the pulp which you would fill in large part with cement to protect the pulp you may protect the tooth, but in making inlays it would be better to carry the matrix down to the cavity and let the porcelain come to the film of cement. When you come to put on those corners, I am inclined to think that the easier way would be just as good. I do not believe there is any need of cutting away solid tooth when putting in an inlay, because the inlay will protect from decay. One of the best features of inlay work is the minimum of taxation of the patient's strength.

*Dr. Smith.*—Do you attribute the immunity to decay to the glazed surface? Do you mean the non-adhesiveness of materials to the surface of the porcelain?

*Dr. Reeves.*—Yes, I think the glazed surface produces the immunity to decay.

*Dr. Smith.*—Between the filling and the cavity margin there is sometimes a small amount of cement washed out. Is there not danger of this causing decay of the proximate tooth?

*Dr. Reeves.*—In the majority of cases such margins do not extend the depth of the enamel. Dr. Ames, of Chicago, thinks this is the reason why it lasts under the inlays when it would wash out in other places. There are lots of things hard to answer, and I do not pretend to explain the fact that we have no decay around inlays. It is certainly a fact that we do not. It is equally true that with the simplest of gold fillings we do have recurrence of decay. I mentioned the glaze upon the inlay surface as the most plausible explanation. We have many amalgam fillings about which there has been no recurrence. When we consider that class of mouths in which teeth decay rapidly and no ordinary fillings last, and find that porcelain does preserve the teeth, it is a pretty strong argument in favor of porcelain. In a case of a young lady coming into my hands in 1892, in which gold had lasted but a year, the porcelain inlays lasted until three years ago. Cement fillings were put in, but I advised replacing the inlays, and they have worn well now for three years.

*Dr. Smith.*—Did you have recurrent decay around the cement fillings?

*Dr. Reeves.*—The contour of the cement fillings had to be renewed. If I had left them, there would have been decay.

*Dr. Smith.*—Would it not be possible that the oxyphosphate of zinc was the immunizing factor? We all know that zinc has antiseptic action. You can see areas around zinc where bacteria will not accumulate.

*Dr. Reeves.*—It may be. I am advancing what I think to be the cause of the immunity.

OTTO E. INGLIS,
*Editor Academy of Stomatology.*

